# Dynamic Programming Based Segmentation in Biomedical Imaging

**DOI:** 10.1016/j.csbj.2017.02.001

**Published:** 2017-02-16

**Authors:** Kathrin Ungru, Xiaoyi Jiang

**Affiliations:** a Department of Mathematics and Computer Science, University of Münster, Münster, Germany; b Cluster of Excellence EXC 1003, Cells in Motion, Münster, Germany

**Keywords:** Dynamic programming, Active contours, Energy minimization, Shortest path, Segmentation, Contour detection

## Abstract

Many applications in biomedical imaging have a demand on automatic detection of lines, contours, or boundaries of bones, organs, vessels, and cells. Aim is to support expert decisions in interactive applications or to include it as part of a processing pipeline for automatic image analysis. Biomedical images often suffer from noisy data and fuzzy edges. Therefore, there is a need for robust methods for contour and line detection. Dynamic programming is a popular technique that satisfies these requirements in many ways. This work gives a brief overview over approaches and applications that utilize dynamic programming to solve problems in the challenging field of biomedical imaging.

## 1 Introduction

Dynamic Programming (DP) introduced by Richard Bellman [1] is a widely used technique to solve optimization problems in a simple and efficient way. In computer vision, Amini et al. [2] showed on the example of active contours how DP can be utilized to perform energy minimization. Furthermore, DP was particularly used to detect lines in images [3] especially in the field of road detection in satellite images for example by Merlet and Zerubia [4], while Buckley and Yang [5] applied DP to solve a shortest path (SP) problem.

In biomedical imaging DP is a popular technique to find contours, lines and boundaries of organs, bones, vessels and cells. This survey focuses on applications in the field of biomedical imaging in particular on the detection and tracking of contours and structures by means of DP.

We organize our work as follows. In Section 2 we motivate this work by giving a short overview of issues in biomedical imaging. Then, in Section 3 we introduce common problems solved by DP and show examples of applications in Section 4. Finally, we conclude our work in Section 5.

## 2 Motivation

In biomedical imaging many computer vision problems involve the detection of objects in pictures acquired through the various types of imaging techniques. A goal is to help physicians to automatically detect, track and analyze structures in biomedical images, to reduce the expert's workload, increase the productivity, and improve the accuracy of the diagnosis.

### 2.1 Application Overview

An example is the detection of the endocardial border of the heart [6], [7], [8], [9] and its movement [10], [11], [12] that gives valuable knowledge (visually and quantitatively) about the heart function. Also artery and vessel boundary detection, e.g. presented in [13], [14], [15], [16], [17], [18], [19], [20], [21] is of great interest, where the detection and evaluation of vessel boundaries and vessel thickness (intima-media) is a marker to detect stenosis [16] or helps in the diagnosis of atherosclerosis [15], [19]. The work in [22] proposes a technique to access the tree of fine vessels to determine their progress and density. Beside investigations in the field of blood supply, biomedical imaging is used to detect every kind of tumors, organs, bones, and even cells. The works in [23], [24], [25], [26] propose methods to detect the ribs, spines and bones or specific parts of the spine, while [27], [28] focus on tumor and cancer detection. The segmentation of microscopic cells includes approaches, where cell borders are detected fully automatically, e.g. [29], [30], [31], or where a single cell is segmented in a preselected ROI as done in [32], [33]. Finally, there are various applications that utilize DP in ophthalmology to examine parts of the eye [34], [35], [36] and in the field of mammography to detect breast cancer [37], [38], [39], [40], [41].

### 2.2 Method Overview

Imaging modalities, e.g. MRI, ultrasound, X-ray, and microscopy, not only differ from their fundamental physical idea, but also in terms of usage (with contrast marker or without; invasive or not), application (2D or 3D; still images or image sequences), and the object or body part of interest. There exist a wide range of techniques and applications to detect and analyze their content. Some applications work totally automatically and some need user interaction. Most of these approaches have to deal with difficulties like inhomogeneities in the intensity of the targeted structures, strong noise or other artifacts depending on the acquisition system.

In terms of the described problems and the demand on a specific robustness, DP draws particular attention in biomedical imaging as it always finds a global optimum and it outputs a connected path despite of the presence of inhomogeneities and holes in the underlying image features. The various studies, introduced in this section, have in common to use DP in numerous ways.

Specific applications evoke specific questions. Most of the reviewed works try to find the shortest path to detect a contour or boundary in the image by minimizing some energy function by means of dynamic programming. Finding a contour or line by DP, for instance in ultrasonic data, demands techniques to properly carve out edges in the presence of noise and artifacts. This requires appropriate filtering and noise reduction such as proposed by Jia et al. and Lee et al. [26], [27] or the integration of high-level information and prior knowledge to overcome uncertainties as approached by Oost et al., Koh et al. and Ungru et al. [9], [23], [42].

Another application with specific requirements are circular objects like cardiac and vascular borders in ultrasound and MRI [10], [11], [13] or cells in microscopic images [29], [31], [32], [33]. Also the detection of the mammographic mass in a preselected ROI [37], [38], [39], [40], [41] or the segmentation of the optic disk in retinal fundus images of the eye as done in [36] aims to find circular structures by means of DP. These applications arise the need of finding a circular path with minimal cost: a circular shortest path (CSP). A CSP beside the optimality constraint demands the closedness of the contour as further restriction and is generally discussed by Sun and Pallottino [43] and Appleton and Sun [44] and applied on biomedical images among others in [8], [10], [11], [13], [24], [29], [33], [37], [38], [39].

The evaluation of vessel border thickness [15], [17], [18], [19], spine boundaries [25], [45], ribs [24], or retinal [35] and corneal layers [34] necessitates to detect structures with two or more nearly parallel contours. In general, the set of simultaneous paths with the lowest cost in total is referred to as multiple shortest path (MSP). Nevertheless, it is important to note that not all of the works above search for an optimal solution for this problem.

A special type of shortest paths are active contours. Active contours are popular in biomedical imaging and can be implemented with DP as shown by Amini et al. [2]. Active contours usually need an initial contour, which is obtained by user interaction, random generation, or a contour of a previous frame (in image sequences). This initial contour is attracted iteratively through some forces to a local minimum as originally proposed by Kass et al. [46]. While active contours by Kass et al. are modeled as continuous curves, Amini et al. introduce a discrete DP-based optimization approach, where contours are represented by some control points connected via splines. A non-iterative approach of deformable contours is proposed in [10], [11] to attract a contour (represented by a few control points) to the left ventricular border in MRI and track it over time. Other approaches like [19], [25] mainly use shape constraints instead of initial points to diminish the search space to arrange the contour points and attract it to an object border. Deformation and tracking over time is also examined in the approach of Pham and Doncescu [28].

A further application of DP in biomedical imaging is proposed in [22], where a vascular tree is detected and represented as a graph by means of a region growing technique based on DP. This approach is the only reviewed approach that is not based on energy minimization.

## 3 Problems and Solutions

As discussed in Section 2 the introduced applications can be categorized into a few problems. Most of them can be summarized as energy minimization tasks. A transfer of these problems into graphs allows us to simplify and generalize the description of the various reviewed approaches. An optimal path, hence the path with the lowest cost in a graph is also known as shortest path. This section gives a brief overview of shortest path problems solved by DP and introduces the most common methods (Table 1).

### 3.1 Solving Shortest Path Problems by Dynamic Programming

A graph is a structure that contains nodes connected by edges. A path in a graph is a connection of several nodes via edges. Each edge can be associated with a specific weight, also known as cost. Then, finding the shortest path in a graph means finding the path with the lowest cost sum of all edges in the path. According to Felzenszwalb and Zabih [47] there are two forms of shortest path problems. The *single-source* type searches the shortest path from a source point *s* to each of the remaining nodes while the *all-pairs* search tries to find the shortest path between each possible pair of nodes in a graph. The mentioned shortest path problems can be solved by generic shortest path algorithms such as proposed by Dijkstra [48]. For an overview we refer to [49].

**Fig. 1:**
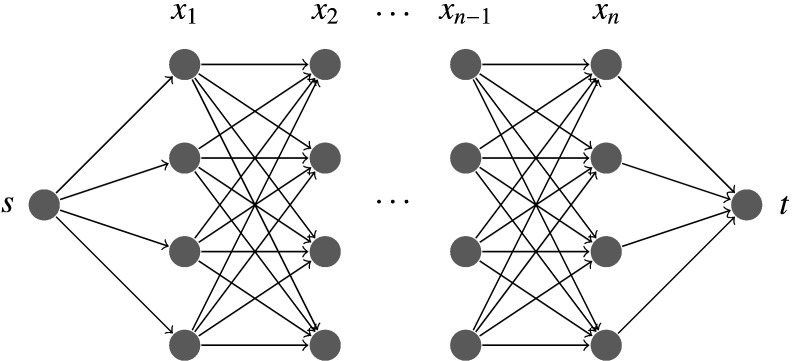
Possible paths $\left(x_{1},x_{2},\ldots ,x_{n}\right)$ in a graph from node *s* to node *t* for the case *k* = 4.

The single-source shortest path is the most frequently used type and can be efficiently solved by DP. Dynamic programming sequentially solves the shortest path problem by splitting it into simpler subproblems. Starting at node *s*, at each state i=1,…,n, the algorithm evaluates the shortest path back to *s*. Because DP works sequentially, it can only find shortest paths in a directed acyclic graph (DAG) that is exemplary illustrated in Fig. 1.

A shortest path search is often utilized for discrete energy minimization as shown in [5]. A common description of energy in computer vision consists of two terms: energy based on observations in some underlying data and energy of some prior, including constraints of smoothness:

(1) $$E=E_{data}+E_{prior}$$

Let $\left(x_{1},x_{2},\ldots ,x_{n}\right)$ be an arbitrary path of *n* elements. Then, the energy of this path is defined as:

$$E=E(x_{1},x_{2},\ldots ,x_{n})$$

The energy of the optimal path $\left(x_{1}^{*},x_{2}^{*},\ldots ,x_{n}^{*}\right)$ is obtained by minimizing *E*:

$$\min(E)=E(x_{1}^{*},x_{2}^{*},\ldots ,x_{n}^{*})$$

In accordance to formula (1) the energy of a discrete path can be described as follows:

(2) $$E(x_{1},x_{2},\ldots ,x_{n})=\overset{n}{\underset{i=1}{\sum }}c(x_{i})+\overset{n}{\underset{i=2}{\sum }}d(x_{i-1},x_{i})$$

where the data term *c*(*x*_*i*_) is the cost of the path passing through *x*_*i*_ and the smoothness term *d*(*x*_*i* −1_,*x*_*i*_) is the cost of the partial path between *x*_*i* −1_ and *x*_*i*_. *c*(*x*_*i*_) can be, e.g., a feature computed on the basis of image intensity data, while *d*(*x*_*i* −1_,*x*_*i*_) is typically a geometrical cost where specific neighborhoods can be penalized in terms of their position to each other, and thus is based on some prior knowledge of the path characteristics.

To perform DP, the energy evaluation of a path is split into simpler subproblems:

$$\begin{array}{c} E(x_{1})=c(x_{1})\\ E(x_{1},x_{2})=E(x_{1})+c(x_{2})+d(x_{1},x_{2})\\ E(x_{1},x_{2},\ldots ,x_{i})=E(x_{1},x_{2},\ldots ,x_{i-1})+c(x_{i})+d(x_{i-1},x_{i})\\ E(x_{1},x_{2},\ldots ,x_{i},\ldots ,x_{n})=E(x_{1},x_{2},\ldots ,x_{i},\ldots ,x_{n-1})+c(x_{n})+d(x_{n-1},x_{n}) \end{array}$$

with 1 ≤ *i* ≤ *n*. The energy at each state is the sum of the preceding energy and the current cost. Now energy minimization by DP is performed with the following recursive formula:

(3) $$\begin{array}{l} C_{1}(x_{1})=c(x_{1})\\ C_{i}(x_{i})=c(x_{i})+\underset{x_{i-1}}{\min}(C_{i-1}(x_{i-1})+d(x_{i-1},x_{i})) \end{array}$$

*C*_*i*_ typically is a table of *k* entries, where each entry *x*_*i*_ stores the minimal cumulative cost of the shortest path from *x*_*i*_ back to the beginning of the graph. Hence, by evaluating the minimum of all cost entries at *C*_*n*_, one can find the starting point of the global shortest path through the entire graph from state *n* back to state 1 by:

$$x_{n}^{*}=\arg\underset{x_{n}}{\min}C_{n}(x_{n})$$

such that for the cumulative cost entry at $x_{n}^{*}$ it holds:

$$C_{n}(x_{n}^{*})=E(x_{1}^{*},x_{2}^{*},\ldots ,x_{n}^{*})=\min(E)$$

Finally, starting with $x_{n}^{*}$ we are able to track back the global shortest path in order of decreasing *i* with:

(4) $$x_{i}^{*}=\arg\underset{x_{i}}{\min}(C_{i}(x_{i})+d(x_{i},x_{i+1}^{*}))$$

The advantages of DP on shortest path problems become noticeable by looking at the processing time of the algorithm. An exhaustive search for the shortest path in the graph visualized in Fig. 1 would yield a complexity of $O(k^{n})$. By applying DP the efficiency increases to $O(k^{2}n)$, as for each of the O(n) tables there are O(k) entries to be filled in, in a time O(k) caused by the minimization of the neighbors. This is more efficient than, for example, to run the generic shortest path algorithm of Dijkstra with $O(k^{2}n\log(kn))$ on this problem. For more details we refer to the work of Felzenszwalb and Zabih [47].

Algorithm 1 Solving shortest path problems in a DAG.

**Figure:**
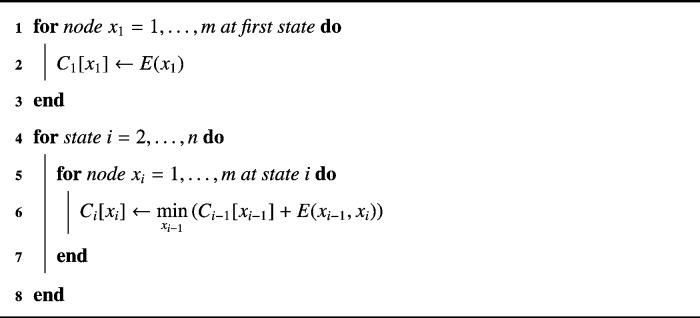

With the previous definitions any DAG can be handled. Also an image matrix or grid can be seen as a graph, where the neighboring pixels are connected. To solve shortest path problems in images it is needed to turn the image graph into a DAG. The two most common techniques are discussed in 3.2, 3.4. Another way of DAG creation is performed by the RACK algorithm proposed by Jiang et al. [50]. In this work a DAG is built based on some skeleton points, set by the user, depicting the skeleton of an object of interest. This method enables the segmentation of objects of any shape via DP. Also the work in [39] introduces an alternative way of DAG construction to compute circular structures in the original image space instead of transforming it into a polar representation (cf. Section 3.3 for more details).

### 3.2 Matrix-Based Shortest Path

**Fig. 2:**
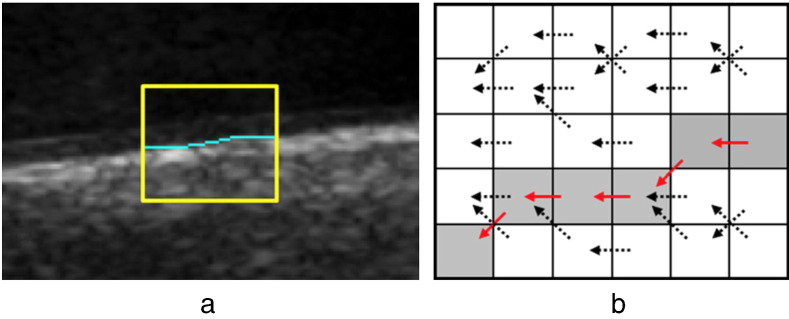
(a) A contour detected in the ROI of a carotid artery image; (b) backtracking process in a cumulative cost matrix as discussed in Section 3.1.

Source: (Reprinted from [17], with permission from Elsevier).

A common application of DP is to find a connected contour (line, boundary) in an *m* × *n* image matrix or grid as shown in Fig. 2. This contour is a shortest path traversing the image from left to right (or top to bottom), where it passes each image column (or row) exactly once. In the following we refer to the type of contours traversing the image from left to right, but the description can also be applied to contours from top to bottom by interchanging rows and columns.

A pixel-node *x*_*i*_ of the path is connected to its neighbors *x*_*i* −1_ in the previous column; most commonly three connected neighbors (*k* = 3). This simplifies Fig. 1 to having each node connected to three predecessors only. Due to the DP process in each column *i* the cumulative cost of each pixel-node *x*_*i*_ is computed sequentially starting in the first column according to formula (3). Hence, each table *C*_*i*_ contains *m* entries of cumulative minimal costs, such that the number of possible starting points for the backtracking procedure in formula (4) equals to the number of rows of the image matrix. Beside a restriction to a specific number of neighbors *k* ≤ *m* the DP process is equal to the shortest path algorithm introduced in the previous section.

In common approaches each pixel, hence node, obtains a cost based on local features. This may be, for instance, the intensity *f*(*x*_*i*_) of a pixel itself or its gradient magnitude to find a line along edges:

$$c(x_{i})=-∥\nabla f(x_{i})∥$$

Note that the sign of the gradient feature must be inverted in terms of energy minimization.

Gradient and intensity based costs suffer from strong noise or fuzzy edges that can lead to ambiguities in the local pixel neighborhood, and thus to a misleading path. To avoid this, another class, named region-based contour detection by DP, is proposed in [32], [33]. Region-based segmentation methods compute their cost based on regional information rather than local features. In contrast to finding a path along the strongest edge, it finds an optimal path that splits two homogeneous regions. The cost of a pixel *x*_*i*_ splitting two regions, say the region above and the region below, according to Jiang and Tenbrinck [32], is computed as follows:

$$c(x_{i})=\underset{x_{\text{above}}}{\sum }∥f(x_{\text{above}})-\mu_{\text{above}}∥+\underset{x_{\text{below}}}{\sum }∥f(x_{\text{below}})-\mu_{\text{below}}∥$$

where ∥⋅∥ can be any valid norm, *x*_*above*_ and *x*_*below*_ are the pixels above and below the current pixel *x*_*i*_, and *μ*_*above*_ and *μ*_*below*_ are simply the averages of these pixels, respectively.

In terms of processing time a matrix-based shortest path solved by DP has to store *O*(*m*) entries into *O*(*n*) tables in *O*(*k*) time and thus has a complexity of O(kmn), that is linear to the image size. An exhaustive search in turn would yield a complexity of $O(k^{n-1}m)$ on this problem. The described methods are illustrated on the example of contours, where the smallest element is a pixel. Instead of pixels one can also take elements of higher order, like segments, that define the curve (see Section 3.4). This may lead to higher robustness and less processing costs, but highly depends on the specific application. Finally, for the detection of 3D surfaces in medical images we refer to [14], where the 2D matrix-based DP algorithm is expanded to detect 3D surfaces. Cheng and Liu [13] point out that 3D DP due to its high complexity is somehow unpractical. To improve this issue they introduce constraints to limit computational time.

### 3.3 Circular Shortest Path

**Fig. 3:**
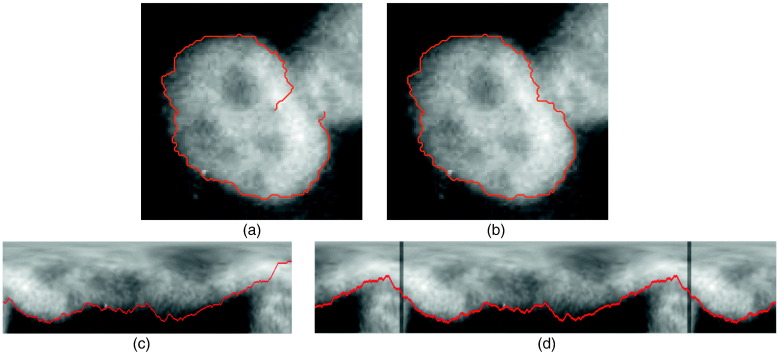
(a) SP of a HEp-2 cell in Cartesian space; (b) CSP of the cell in Cartesian space; (c) SP in polar space; (d) CSP in polar space evaluated with IPA.

Source: (HEp-2 cell data from [51]).

A CSP is a special form of a shortest path with the restriction that start and end nodes are connected. One can imagine applications in 360° panoramic or surface images, or finding circular structures in images. In biomedical applications the circular structures can be masses in mammography, the endocardial border, or cells in microscopic images (cf. Fig. 3), but also body surface images, e.g. of ribs, that have the same start and end points on the left and right side of a projected 2D surface map [24] (cf. Fig. 7).

Circular structures like cells or the cardiac border often are detected by transforming it into a polar representation, where each ray around a center point in the middle of the structure depicts a column in the polar space. Then, the circular contour corresponds to a contour from left to right as discussed in Section 3.2 and illustrated in Fig. 3 (c).

**Fig. 4:**
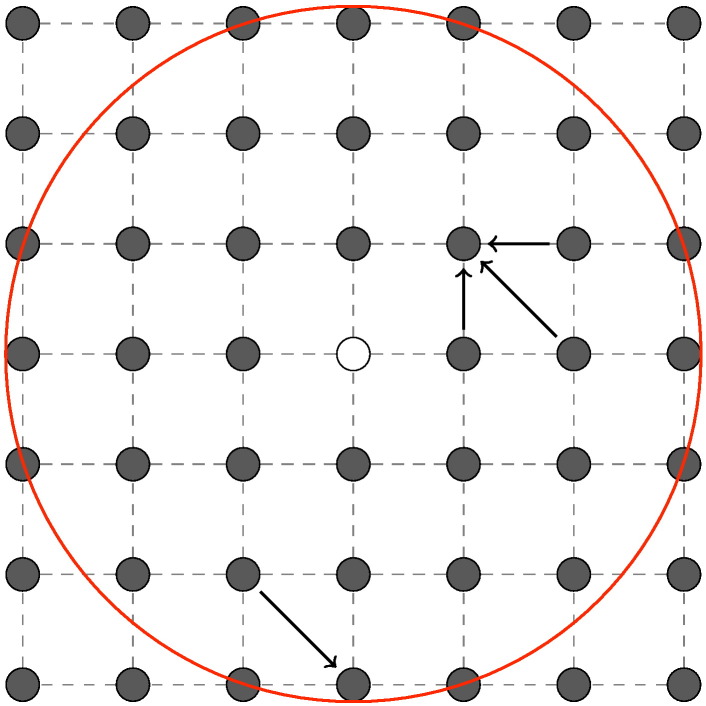
Some example nodes and their adjacent neighbors in a DAG ordered by the polar angle.

Another possibility of finding circular structures in images is proposed in [39]. Instead of converting the image space into a polar representation that may yield interpolation issues, the authors create a DAG based on the polar coordinates of the pixel-nodes. Through this approach, optimization is not as before evaluated in relation to the nodes in the preceding column, but to the nodes in the 8-neighborhood that have a lower polar angle. Hence, ordering factor to meet the sequential nature of DP in this case is not given through the *x* coordinate (column) of a node in Cartesian space but rather through the polar coordinate of the nodes with respect to a predefined center (cf. Fig. 4).

The circularity in both methods is achieved by constraining the closedness of the contour. Note that only convex and star-shaped contours^1^ can be detected in both cases. Several CSP strategies are examined on the example of matrix-based DP by Sun and Pallotini [43] and Appleton and Sun [44] to constrain the closedness. The goal is to find a contour in an *m* × *n* matrix or grid supposing the matrix were wrapped onto a cylinder and hence the first and last columns of the matrix are neighbors. Not all of these algorithms guarantee to find the globally optimal CSP or even any circular path. But, according to Sun and Pallotini [43] the algorithms can be combined to reach a certain accuracy or at least the closedness of the result. In the following we briefly discuss the basic ideas.

#### 3.3.1 Multiple Search (MSA)

The multiple search algorithm is a straightforward method that gives a guarantee of closedness and optimality. The algorithm selects the first node in the first column and determines the corresponding neighbors in the last column. All nodes in the first and last column are set to a very high cost value except of the selected nodes that keep their costs. After this initialization phase, the matrix-based DP is performed. This is repeated for each node in the first column, thus *m*-times. Finally, the minimal cost path is evaluated out of the *m* candidates. This algorithm has a complexity of $O(km^{2}n)$ as it runs *m*-times the matrix-based DP algorithm.

#### 3.3.2 Image Patching (IPA)

The image patching algorithm is a simple and fast method, where the image size is extended by copying a patch, meaning a specified number of columns on the left, onto the right side of the image and vice versa (cf. Fig. 3 (d)). This attracts the contour detection to circularity, but does not give a guarantee of closedness. As it just performs the DP optimization once, the algorithm runs in $O(km\hat{n})$, where $\hat{n}>n$ is the size of the extended image. To force the algorithm to be closed, it can be repeated with different patch sizes.

#### 3.3.3 Multiple Backtracking (MBTA)

The multiple backtracking algorithm, as the name says, simply tracks all path candidates in the last column back to the first column (instead of backtracking the one with the lowest minimal cost). If the nodes in the first and last column of a path candidate are neighbors, the found path is a possible candidate for the CSP. After this step, again the minimum of all possible candidates is the resulting CSP. While Sun and Pallotini just state that this algorithm has a high probability to find a CSP, Malon and Cosatto [33] and Cardoso et al. [39] prove that this algorithm actually guarantees to find at least one solution for this problem. The complexity is the same as the complexity of the matrix-based algorithm O(kmn).

#### 3.3.4 Branch and Bound

A technique is proposed in Appleton and Sun [44] to perform a branch and bound method. Therefore a lower bound is evaluated by performing the matrix-based DP algorithm once without the closedness constraint. The cost of the found shortest path serves as lower bound to decrease the search space for the circular shortest path.

### 3.4 Active Contours by Dynamic Programming

We briefly mentioned before that contours must not necessarily consist of pixels. They can also be composed by segments or some control points that define a curve by spline or polygon approximation. This leads us to another popular class of contour detection approaches, the active contours. As mentioned before, active contour models (or snakes) were originally introduced by Kass et al. [46] as continuous curves that are initialized for example by user interaction and iteratively attracted through some internal and external forces to edges or pixel intensities in an image. The attraction process is performed with the help of generic energy minimization methods. Later on, the work of Amini et al. [2] showed the possibilities of energy minimization via DP on the example of active contours.

An active contour according to Amini et al. is defined by *n* control points that are connected via splines. Each control point of an initial contour has *k* possibilities in its local pixel neighborhood to move to. This again is covered by the graph in Fig. 1, as it is a shortest path problem as described in Section 3.1, where each pixel in the neighborhood of control point *i* denotes a node in the graph at state *i*.

The energy definition is similar however a little extended to the definition in formula (1):

(5) $$E=\underset{E_{ext}}{\underset{︸}{E_{data}+E_{con}}}+\underset{E_{int}}{\underset{︸}{E_{prior}}}$$

The internal energy *E*_*int*_ is identical to the smoothing term *E*_*prior*_. The external energy *E*_*ext*_ contains the energy based on the image data (*E*_*data*_) and the energy of a hard constraint *E*_*con*_ = −*λ*|*x*_*a*_ − *x*_*b*_| that is a spring-like cost between a point on the curve and a point in the image. This cost gives the possibility to force the curve in direction to some user defined points outside the initial contour. In the discrete case the total energy is defined as follows:

(6) $$E(x_{1},x_{2},\ldots ,x_{n})=\underset{E_{ext}}{\underset{︸}{\overset{n}{\underset{i=1}{\sum }}c(x_{i})}}+\underset{E_{int}}{\underset{︸}{\overset{n}{\underset{i=2}{\sum }}d(x_{i-1},x_{i})+\overset{n-1}{\underset{i=2}{\sum }}e(x_{i-1},x_{i},x_{i+1})}}$$

where *c*(*x*_*i*_) contains hard constraints and data costs based on local intensity or gradient features (or a combination of both). The smoothing terms *d*(*x*_*i* −1_,*x*_*i*_) and *e*(*x*_*i* −1_,*x*_*i*_,*x*_*i* +1_) are specified as follows:

$$\begin{array}{c} d(x_{i-1},x_{i}):=\alpha \;|x_{i-1}-x_{i}{|}^{2}\\ e(x_{i-1},x_{i},x_{i+1}):=\beta \;|x_{i-1}-2x_{i}+x_{i+1}{|}^{2} \end{array}$$

The first order term *d* favors the points to become closer to one another and the second order thin-plate term *e* favors the points to become equidistant.

The second order term *e* needs a point *x*_*i* +1_ that is not yet available at state *i* in the ordinary DP. Therefore, Amini et al. [2] propose an adjustment called “time-delayed” DP. For each combination (*x*_*i*_,*x*_*i* +1_) all *k* neighbors *x*_*i* −1_ are determined and the cost *e*(*x*_*i* −1_,*x*_*i*_,*x*_*i* +1_) is evaluated such that for the optimization step according to formula (3) by means of DP applies:

(7) $$\begin{array}{l} C_{1}(x_{1},x_{2})=c(x_{1})+d(x_{1},x_{2})\\ C_{i}(x_{i},x_{i+1})=c(x_{i})+d(x_{i},x_{i+1})+\underset{x_{i-1}}{\min}\left(C_{i-1}(x_{i-1},x_{i})+e(x_{i-1},x_{i},x_{i+1})\right) \end{array}$$

Note that this step multiplies the amount of entries in table *C*_*i*_ by *k* as there exist *k* × *k* possible combinations of (*x*_*i*_,*x*_*i* +1_) at each state of the DP process. After computing the cumulative minimal cost values at each state (control point), the backtracking process is performed according to:

(8) $$x_{i}^{*}=\arg\underset{x_{i}}{\min}\left(C_{i}(x_{i},x_{i+1}^{*})+e(x_{i},x_{i+1}^{*},x_{i+2}^{*})\right)$$

To obtain a final developed contour the described DP process is to be repeated iteratively as long as the energy converges.

The complexity of the contour approach in comparison to the ordinary algorithm in Section 3.1 increases from $O(k^{2}n)$ to $O(k^{3}n)$. At each of the O(n) control points a table of size $O(k^{2})$ of possible node combinations is to be written according to the number of neighbors *x*_*i* −1_ of the combination (*x*_*i*_,*x*_*i* +1_). The number of iterations additionally multiplies the computational cost. Finally, it is to be mentioned that a high amount of *k* pixels in the neighborhood of a control point indeed increases the search space around a control point and hence the globality of the result, but comes with an increase of complexity. This is to be considered by selecting an appropriate value for *k*.

Algorithm 2 One iteration of the active contours algorithm by DP.

**Figure:**
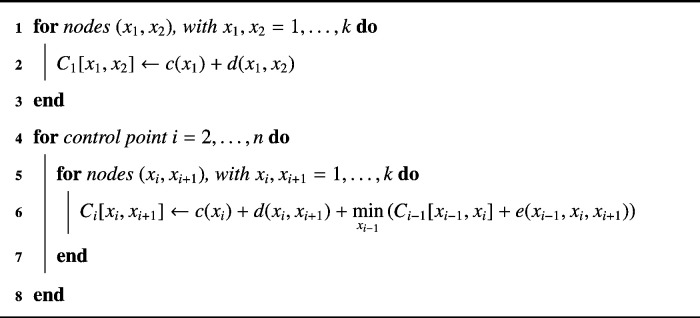

Realizing a circular active contour means to handle the first and last control point as neighbors. This would imply a circular graph with smoothness costs *d*(*x*_*n*_,*x*_1_), *e*(*x*_*n* −1_,*x*_*n*_,*x*_1_) etc., that is in turn not solvable by DP. One can overcome this problem by fixing two neighborhood points (nodes) around the first and the last control point, respectively. Similar to the MSA variant in Section 3.3, all possible pairs of nodes are to be tested, which increases the complexity to $O(k^{4}n)$. The high processing cost in terms of active contours can be overcome, according to Felzenszwalb and Zabih [47], by randomly fixing two neighboring nodes at each iterative step. This keeps the contour close, but still lets the curve converge against a minimum over time with an ordinary complexity of $O(k^{3}n)$.

### 3.5 Multiple Shortest Path

**Fig. 5:**
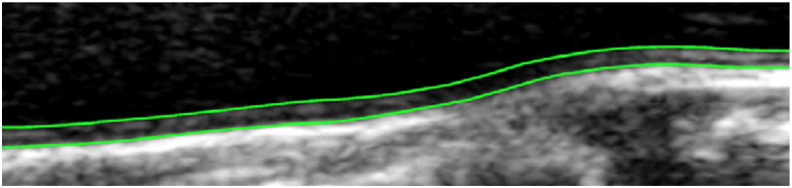
Detection of intima-media thickness in ultrasound carotid artery image.

Source: (Reprinted from [19], with permission from Elsevier).

Finally, the discussed methods (matrix-based shortest path, active contours) can be extended to a multiple shortest path approach as examined for example in [15], [19], see Fig. 5 for an illustration. Let *p* be the amount of simultaneously detectable paths. This means that there must exist at least *p* directed acyclic graphs of the same structure. Each of the *p* paths in the graphs consists of *n* nodes. The authors of [19] use a general vector notation to describe the parallel nodes $x_{i}={\left(x_{i,1},x_{i,2},\ldots ,x_{i,p}\right)}^{T}$ at each state in the optimal path search. This results in the following general definition for *p* simultaneous contours by extending the energy definition in formula (1):

(9) $$E(x_{1},x_{2},\ldots ,x_{n})=\overset{n}{\underset{i=1}{\sum }}c(x_{i})+\overset{n}{\underset{i=2}{\sum }}d(x_{i-1},x_{i})$$

Accordingly, to the definition of ordinary DP in formula (3) for MSP applies:

(10) $$\begin{array}{l} C_{1}(x_{1})=c(x_{1})\\ C_{i}(x_{i})=c(x_{i})+\min_{x_{i-1}}(C_{i-1}(x_{i-1})+d(x_{i-1},x_{i})) \end{array}$$

and the backtracking starting at *i* = *n* is represented by:

(11) $$x_{i}^{*}=\arg\min_{x_{i}}(C_{i}(x_{i})+d(x_{i},x_{i+1}^{*}))$$

This looks very similar to the single path optimization by DP, where the data cost term is often defined as the sum of the single contour costs $c(x_{i})=\sum_{t=1}^{p}c(x_{i,t})$. The smoothing term *d*(**x**_**i −1**_,**x**_**i**_) in this definition includes more than just the smoothness within the single curves, which is called *intra-curve* smoothness. It also includes *inter-curve* constraints considering the behavior between the multiple curves including a specified minimal distance to each other.

As an example the approach in [15] defines the following terms $c(x_{i})=\sum_{t=1}^{p}G(x_{i,t})$ and *d*(**x**_**i −1**_,**x**_**i**_) = *α*|*D*(**x**_**i −1**_) − *D*(**x**_**i**_)| + *β*||**x**_**i −1**_ −**x**_**i**_∥_1_ with *p* = 2 to realize the algorithm, where ∥⋅∥_1_ denotes the *L*_1_-norm and *D*(**x**_**i**_) = *x*_*i*,2_ − *x*_*i*,1_. Data costs are computed with the help of a gradient edge filter *G*(⋅). Additionally, to guarantee a specific distance between the two curves and to avoid overlapping, the following must be valid for all opposing points on the two lines *D*_*min*_ ≤ *D*(**x**_**i**_) ≤ *D*_*max*_.

The most considerable difference to a single path approach, despite of the smoothness term, is the increase of complexity. Considering a dual path approach with *p* = 2 and *k* = 3 neighbors without any constraints, there are 9 possible combinations of preceding node pairs to the current pair of nodes. In general in case of matrix-based MSP this turns out to a complexity of $O(k^{p}m^{p}n)$, as there are $O(m^{p})$ possible pairs of nodes in a column, that have $O(k^{p})$ preceding neighbors to be optimized. The dramatical increase of processing time in dependence to *p* shows the importance of constraints to reduce the search space.

### 3.6 Region Growing for Vessel Tree Detection

**Fig. 6:**
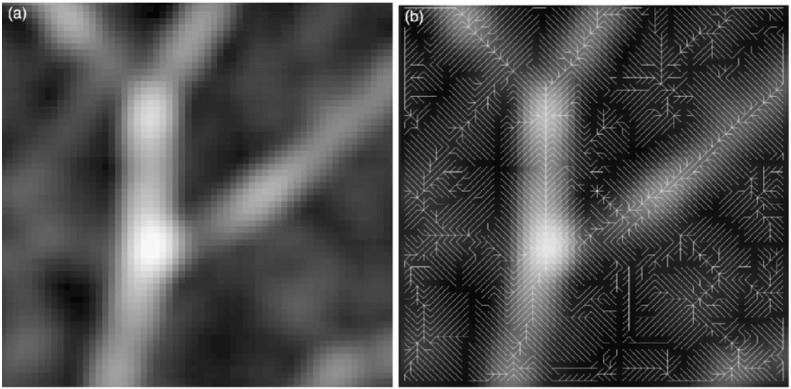
Detection of the vessel tree; (a) original image; (b) entire vessel tree retrieved by the ORG algorithm.

Source: (Reprinted from [22], with permission from Elsevier).

As last method in this section we present a region growing algorithm based on DP proposed in [22]. It is the only technique that does not minimize energy and is in this form method and application at once. The detection of vessels refers to two problems: a segmentation problem and a centerline detection problem. In [22] a centerline detection is proposed which is particularly approached to the detection of small vessels. The DP algorithms reviewed in the previous sections build a tree of possible optimal paths. This characteristic of DP is utilized in [22] to build an acyclic graph that connects the entire pixels in the image (cf. Fig. 6) by a method called ordered region growing (ORG). The proposed ORG algorithm is classified as DP, as there exist two characteristic steps: a recursion step and a backtracking step. The ORG algorithm works with sets of pixels, where each pixel is a node in the graph. The graph connectivity is found by setting a seed point *x*_1_, e.g. by user interaction, and by setting the initial region to $R(x_{1})=\{x_{1}\}$. A region grows by adding the neighboring pixels $N(x_{i})$ (8-connected neighbors in 2D) of the seed *x*_*i*_ in iteration *i*. A seed *x*_*i*_ of a region $R(x_{i})$ is found by maximizing the intensity values of the border pixels *I*_*B*_ of the previous region $R(x_{i-1})$:

$$\begin{array}{c} x_{i}=max(I_{B}(R(x_{i-1}))\\ R(x_{i})=R(x_{i-1})\cup N(x_{i}) \end{array}$$

A new edge of the graph is then created by connecting the found seed to each of its neighbors:

$$E_{i+1}=E_{i}\cup \{x_{i},n_{i}\}$$

where $n_{i}\in N(x_{i})$.

The region growing step by adding the neighbors of some optimal seed point can be seen as the recursive accumulation process in DP. The backtracking now is performed to examine the vessel tree. By selecting two nodes in the graph, one can track back the path from one to the other. This path depicts, for instance, a vessel of interest. Finally, Yim et al. [22] propose a trimming procedure to improve the found graph and delete irrelevant branches.

## 4 Applications

In Section 3 we have given a general overview of common problems and their solutions by utilizing dynamic programming. This section summarizes the reviewed papers and analyzes their applications according to the previously presented methods.

In Section 3.2 we described a matrix-based shortest path algorithm that, in the most common form, detects contours based on image gradients, thus requires relatively strong edges. It is noticeable that this typical approach is applied to image modalities with comparatively low noise and high contrast, e.g. MRI [6], [8], [16], [23]. Contour detection in this case is part of a processing pipeline that aims for high-level decisions such as, e.g., stenosis detection in the work of [16]. Here, matrix-based DP is used to refine a rough segmentation of vessels in 3D obtained by a 3D region growing algorithm. The refinement by DP is performed slice by slice inside a specific search area initialized by the result of the previous segmentation. The diameter of the cross-section of the vessel is then used to get information about potentially constricted vessels.

In contrast to MRI, ultrasonic data often suffers from fuzzy edges and strong noise like speckle. Hence, a filtering system is proposed by Lee et al. [27] to enhance edges in ultrasonic images with high presence of scatters, speckle and other artifacts. To suppress strong noise by filtering techniques it is needed to select a relatively large filter kernel. The disadvantage is the potential loss of details. Gradient-based techniques work on local features so that a path can be misled through ambiguities in a local pixel neighborhood. Also microscopic cell images often suffer from weak edges, such as from inhomogeneities in the cell nuclei. To overcome this problem region-based contour detection by DP based on a regional splitting cost is used in [32], [33] (cf. Section 3.2) rather than local edge data. Particularly, a generic framework is proposed in [32] to compute the splitting cost in various ways.

Other works try to overcome noise and artifacts by proposing a coarse segment-based contour and accurate shape constraints. A technique is proposed in [25] to simultaneously detect the left and right spine boundary described by piece-wise linear segments. The DP algorithm hence is not performed pixel-wise, but segment-wise while the gradient-based cost calculation is done by accumulating the cost of the pixels inbetween the end-points of the segments, such that the contour search is not limited by the local pixel neighborhood of the segment's end-points but rather by a more regional cost computation. The shape of the two nearly parallel contours on the one hand is constrained by the contour progress itself (intra-curve constraints) and the two contours interacting with each other (inter-curve constraints), likewise described in the previous Section 3.5. The smoothing constraints of [25] contain constraints of first *d*(*x*_*i* −1_,*x*_*i*_) and second order *e*(*x*_*i* −1_,*x*_*i*_,*x*_*i* +1_). The second order smoothing term is not available in the classical DP algorithm as mentioned in Section 3.4. Hence, to avoid higher complexity the work in [25] does not incorporate the smoothness cost into the DP approach, but proposes an adjusted backtracking process to do so.

The authors of [15] advance the approach of [25], [45] by integrating the before mentioned smoothness term into the dual DP algorithm, but avoid the computation of second order smoothness. The goal of the work is to detect arterial walls in ultrasonic artery images to measure intima-media thickness. Therefore, two contours limiting the intima-media above and below are detected by dual DP. While Cheng and Jiang, and Wei et al. [15], [45] basically try to fit a line against the strongest image edges, the work in [19] goes a step further and proposes to perform the line fitting with the help of a Hough transform. The cost evaluation is performed very similar to the ordinary Hough accumulation process. Smoothness parameters again manage angular and spacial distances in the form of inter- and intra-curve constraints, where a spring-like intra-curve constraint is necessary to handle the connectivity of the single line segments.

**Fig. 7:**
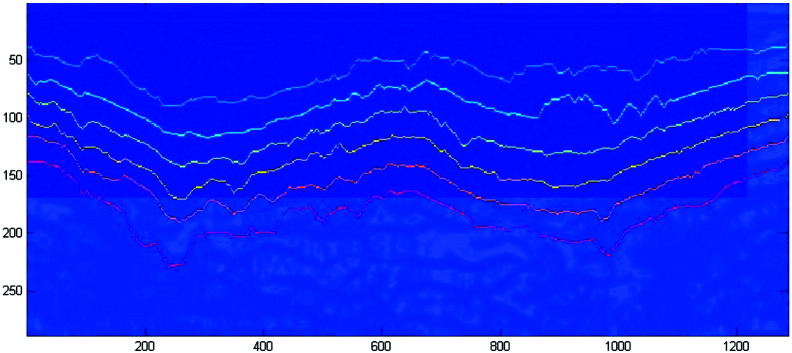
Rib detection in a 2D surface map.

Source: (Reprinted from [24], with permission of Springer).

A rib detection method is proposed in [24] in abdominal 3D MRI based on a multiple contour approach and a circular shortest path solution. In several steps a 2D cost matrix is generated out of the 3D data and a 2D surface map shown in Fig. 7. Seven ribs and hence seven contours are detected from left to right. This is done slightly different from the previous multiple contour methods by computing seven cumulative cost matrices independently and including a distance penalty into the backtracking of the DP algorithm. This encloses a minimal distance constraint between the seven simultaneous paths and avoids the increase of complexity as the algorithm runs in O(pkmn) instead of $O(k^{p}m^{p}n)$ (here *p* = 7) by accepting the loss of global optimality.

As the rib detection is based on an enfolded surface, the rib contours are circular. To guarantee the closedness of the contours, the authors suggest to run the algorithm a second time. In this second run, the found path is used as prior knowledge to guarantee the closedness, such that the actual node is only connected to the preceding node if it leads back to the starting point known from the precalculated path. Finally, it has to be mentioned that although this algorithm finds multiple circular paths with a specific minimal distance to each other, it is not guaranteed to find the optimal global solution.

Further applications for CSP search are microscopic cells as shown in [29] that applies a matrix-based contour detection to oval-shaped objects exemplified on yeast cells and a circular path algorithm based on Sun and Pallotini [43] and discussed in Section 3.3. Also Malon and Cosatto [33] implement a closed contour approach that resembles the multiple backtracking algorithm (MBTA) of Sun and Pallotini, the work in [30] segments bone marrow cells based on a method used in [38], where the weights of the various cost components are evaluated in a training process. Both works are based on an approach in [37] who examine DP to detect masses in computer aided mammography. Timp and Karssemeijer [37] utilize the image patching algorithm (IPA) to compute a closed contour. The optimal patch size here is evaluated heuristically by slightly increasing the patch size and evaluating the number of closed contours. An optimal patch size according to Timp and Karssemeijer [37] is the one, where all found shortest paths are circular.

An example for works that consider closedness in the application of cardiac images is [8] that compares the multiple search method with a branch-and-bound approach applied to myocardial border detection.

Also the work in [10] is mainly devoted to cardiac MR images and discusses a closedness constraint. The authors introduce DP as a tool for detecting, matching and tracking deformable contours in medical images. The approach can be categorized to active contours although it is non-iterative and searches a continuous eight-connected path rather than a set of control points as illustrated in Section 3.4. Nevertheless, the initialization phase is similar, while *n* characteristic points are to be selected to determine a search window and to restrict the DP contour search. The work in [10] utilizes a multi-scale technique to achieve greater processing efficiency while sacrificing guaranteed optimality. The authors proposed this algorithm to detect contours and to track them over time. For the tracking process specific points of high curvature are evaluated from the previous contour and taken as initial control points for the next contour detection. Thereby it is assumed that the movement between the subsequent frames is small and the new contour can be found inside the defined search window. For further examples of interactive organ and bone segmentation in biomedical images we refer to [28], [52].

There are further applications of DP where, in comparison to the applications above, DP is not the essential method to perform a segmentation task. Moreover these applications utilize DP to optimize a segmentation result on a higher level. Hence, an automatic detection of the left ventricular wall is proposed in [9] by using high-level features leaned on experts knowledge like shape, texture, and contraction dynamics to train an active appearance model (AAM) [53] on the basis of manually marked contours. However, the AAM is not incorporated into DP, but taken to perform a rough segmentation to define a search space for the subsequent DP process. DP itself in this algorithm is computed on low-level features based on the intensity values inside the predefined search window.

The work of [7] is an example that uses high-level information to detect the cardiac boundary in 3D ultrasonic data. In this work a 3D shape prior is trained to be fitted via DP to a slice of the ultrasonic heart data. The contour detection itself is then likewise done via DP by matching a trained texture pattern with the help of the previously fitted shape model.

## 5 Conclusion

Most of the reviewed approaches are hybrids of the methods discussed in Section 3. The main differences lie in the computation of cost and the application of different smoothness constraints. Furthermore, lack of a possibility to integrate global shape priors into the DP process, several approaches search for possibilities to constrain their algorithms according to a-priori knowledge based on the application. These constraints typically refer to geometrical characteristics of the observed objects or structures. Hence, for many approaches parameterization is essential. We gave one example of incorporating high-level information by applying DP. Thus, introducing high-level features might be a field of investigations in future. Furthermore, we see a demand in generally incorporating higher order smoothness constraints into the DP algorithm as this is often mentioned but seldom implemented. Another issue, which is of particular interest in medical image analysis, is that of noise modeling [54], [55]. Many existing image segmentation methods (implicitly) assume signal-independent additive Gaussian noise and hence their application leads to suboptimal results, e.g. for ultrasound imaging, which is subject to multiplicative speckle noise. It will be useful to integrate such noise models into dynamic programming based active contours [2] and region-based contour detection by dynamic programming [32].

As a last remark we point out that dynamic programming is limited to optimally detecting 2D contours only. The same optimization problem in 3D space is related to segmenting smooth surfaces in 3D volume datasets or detecting smooth contours in videos. There is no direct way of extending the DP solution to the general 3D case (without cost explosion). Fortunately, efficient solutions do exist for this problem [56], [57], which have resulted in numerous applications.

## Figures and Tables

**Table 1:** Efficiency of the main methods.

Method	
SP	$O(k^{2}n)$
Matrix-based approaches	
– SP	O(kmn)
– MSP	$O(k^{p}m^{p}n)$
– CSP - MSA	$O(km^{2}n)$
– CSP - IPA	$O(km\hat{n})$, $\hat{n}>n$
– CSP - MBTA	O(kmn)
Active contours	
– ordinary approach	$O(k^{3}n)$
– circular contours, MSA	$O(k^{4}n)$
– circular contours, randomly fixed neighbors	$O(k^{3}n)$

## Footnotes

1 A star-shaped contour is characterized by the existence of a point *p* such that for each point *q* of the contour the segment *pq* lies entirely within the contour.
